# Combined and interaction effects of outdoor and reading time on myopia onset: evidence from over 210 000 schoolchildren in China

**DOI:** 10.7189/jogh.15.04274

**Published:** 2025-10-10

**Authors:** Jing Li, Shengxin Liu, Ying Zhu, Desheng Song, Nan Jin, Chunyu Tang, Xuan Li, Bo Zhang, Jiarui Su, Ruihua Wei, Bei Du

**Affiliations:** 1 Tianjin Key Laboratory of Retinal Functions and Diseases, Tianjin Branch of National Clinical Research Center for Ocular Disease, Eye Institute and School of Optometry, Tianjin Medical University Eye Hospital, Tianjin, China; 2 Tianjin University of Tradition Chinese Medicine, Tianjin, China; 3 Tianjin Medical University, Tianjin, China

## Abstract

**Background:**

While numerous studies have examined the individual effects of outdoor and reading time on myopia, the interaction between these factors in large populations remains underexplored. We hypothesised that increased outdoor exposure may mitigate the risk of myopia associated with prolonged reading.

**Methods:**

We included 219 802 initially non-myopic primary school students in the Tianjin Child and Adolescent Research of Eye study. We collected the behaviour exposure data via a questionnaire. We used Cox proportional hazard models, with semesters since entrance as the time scale, to assess the associations of outdoor and reading time with the onset of myopia. We evaluated interaction effects on the additive scale using a generalised propensity score-weighted Cox model, and quantified them by excess relative risk due to interaction (RERI), attributable proportion (AP), and synergy index (SI). Additionally, we conducted subgroup analyses to assess the robustness and consistency of the results.

**Results:**

Among the 219 802 children, 130 246 developed myopia, resulting in a cumulative incidence of 59.3% over three years. Increased outdoor time of 1–2 hours (hazard ratio (HR) = 0.97; 95% confidence interval (CI) = 0.95, 0.98), 2–3 hours (HR = 0.90; 95% CI = 0.88, 0.92), and >3 hours (HR = 0.86; 95% CI = 0.84, 0.89) were associated with a reduced risk of myopia onset, whereas prolonged reading time of 1–2 hours (HR = 1.02; 95% CI = 1.01, 1.03), >2 hours (HR = 1.03; 95% CI = 1.01, 1.05), and >3 hours (HR = 1.05; 95% CI = 1.03, 1.08) were linked to an increased risk. The interaction effects on the additive scale were significant between outdoor time and reading time with RERI of −0.04 (95% CI = –0.08, 0.00), AP of −0.04 (95% CI = −0.07, 0.00), and SI of 0.71 (95% CI = 0.50, −0.93), particularly in boys, those in grades from 1 to 3, and those with myopic parents.

**Conclusions:**

Outdoor time and reading time are not only independently related to the onset of myopia but also interact with each other. These findings highlight the importance of promoting outdoor time and managing reading time to inform targeted myopia prevention strategies.

Myopia has become a critical global public health issue due to its growing prevalence. By 2050, there will be 4758 million people with myopia, accounting for 49.8% of the world population [1]. The rising prevalence poses a severe socioeconomic burden and adversely affects quality of life [2–4]. Previous studies have shown the strong associations between myopia and individual effects of time spent outdoors and near work [5-7]. Digital devices, near-distance reading and other close-up tasks have been associated with an increased risk of myopia, although the evidence level varied. In contrast, outdoor time has been identified as a protective factor against the onset and progression of myopia, as evidenced by several clinical trials [8,9]. However, the underlying mechanisms remain incompletely understood. Proposed factors include higher light intensities, variations in the spectral composition of light, differences in corneal topography, less near work, and decreased accommodative demand [10].

Despite the extensive exploration of the effects of outdoor time and reading time in isolation, relatively few studies have examined the interactive effects between them. It remains unclear whether the benefits of outdoor exposure can offset the risks posed by prolonged reading, and to what extent the relationship between these variables may be synergistic, antagonistic, or independent. Moreover, most existing models do not incorporate interaction terms to statistically evaluate how these behaviours jointly influence myopia outcomes. As a result, our understanding of the behavioural ecology of myopia development remains incomplete. Failing to address the interaction would result in missing the optimal opportunity to prevent myopia.

We aim to address this critical gap by investigating the interaction between outdoor time and reading time among almost 200 000 primary school students from the Tianjin Child and Adolescent Research of Eye study. We aim to quantify whether excess outdoor time could mitigate the effects of prolonged reading time, and whether their interaction exhibits non-additive myopia risk. Our findings may inform more refined public health interventions that balance academic demands with vision-preserving behaviours.

## METHODS

### Study design and participants

The Tianjin Child and Adolescent Research of Eye is a school-based, cohort study initiated in the first half of 2021, supported by the Tianjin government, to promote students’ ocular health. All participants provided a written informed consent form, signed by their parents or guardians, in accordance with the Declaration of Helsinki. The participants were school-age children from a school-based registration system and studying at primary schools in each district of Tianjin, China.

**Figure 1:**
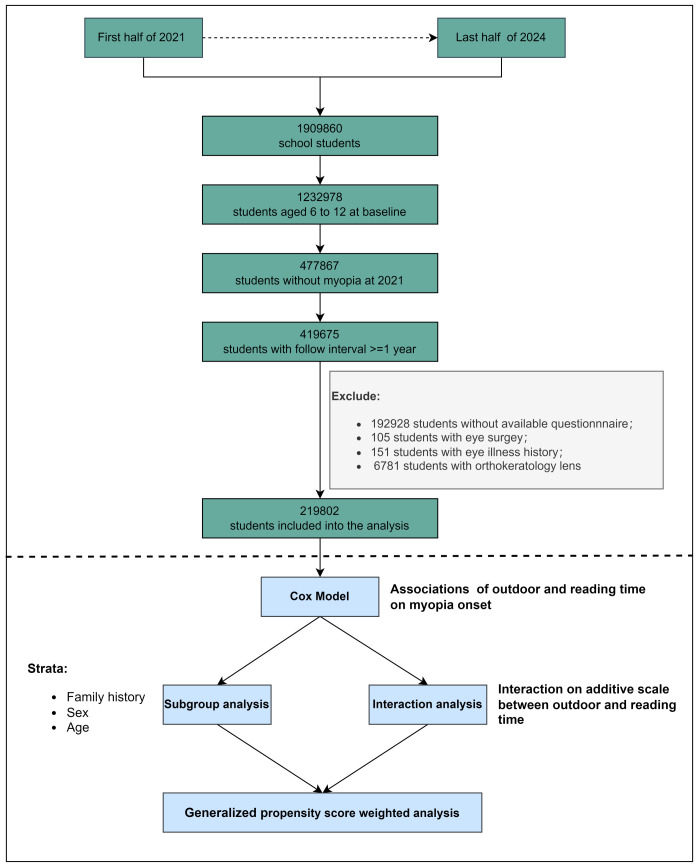
The flowchart of participants and analysis methods.

Between 2021–24, over one million school students participated in myopia screenings. The study initially included 477 867 primary school students without myopia who underwent screening in 2021. However, we excluded 58 192 students who only participated in the 2021 screening and 192 928 students without available environmental data, resulting in a final sample of 219 802 students (**Figure 1**).

### Refraction screening and definition of myopia

In this study, trained ophthalmologists and certified orthoptists used non-cycloplegic refraction (Tianle RM-9600, Shanghai, China) due to practical considerations and the screening setting. Cycloplegic refraction, although more accurate in the paediatric population, is time-consuming, requires medical personnel, and may lead to discomfort or side effects, making it less feasible for large-scale screening. In every exam, they took three repeated measurements and averaged them for statistical analysis. They asked students who wore spectacles or contact lenses to remove them before the examination.

We calculated the spherical equivalent refraction (SER) as the sum of the sphere power and half of the cylinder power. We defined myopia as SER of≤−0.50 D and uncorrected visual acuity of <5.0. Due to the strong correlation between SER values in the two eyes (*r* = 0.899; *P* < 0.001), we used the SER of the right eye as the primary measure for assessing myopia development. We defined incident myopia as myopia that was absent at baseline but present during the subsequent visit.

### Questionnaire and covariates

We collected environmental exposure data via a structured online questionnaire completed by students’ guardians. To ensure data validity, we recorded the completion time for each questionnaire. Response completed in an unreasonably short time, or those that failed random manual quality checks were excluded from subsequent analyses. The information obtained from online questionnaires included demographic characteristics (*i.e.* age, sex, height, weight, and parents’ myopia) and behavioural factors (*i.e.* time spent on entertainment electronic devices (0–1, 1–2, 2–3, and >3 hours), time spent on learning electronic devices (0–1, 1–2, 2–3, and >3 hours), and weekly frequencies of dietary nutrition intake (coarse grain, sea food, fruits, vegetables, snacks, fried foods, carbonated drinks and salt)).

To account for the potential urban-rural disparities in myopia, we categorised the students’ residential areas into six central urban districts, four surrounding urban districts, five suburban counties, and the Binhai New Area.

### Exposure assessment

We primarily assessed exposure by the amount of outdoor time and reading time per day, both obtained from the online questionnaire, with response categories of 0–1, 1–2, 2–3, and >3 hours. We also recorded these as continuous variables by assigning the midpoint value of each interval when referring to the difference between reading time and outdoor time. Additionally, to evaluate the additive interaction, we also classified these variables into two categories: 0–2 and >2 hours, as determined by the maximally selected rank statistics.

### Statistical analysis

There was no variable with >10% missing data. We described continuous data as means (standard deviations (SDs)), and categorical data as numbers (%). To compare groups, we used unpaired, two-tailed *t* tests or Wilcoxon’s rank-sum test for continuous variables, as appropriate, and χ^2^ test for categorical variables.

We used semesters from the entrance into grade one as the timescale in the subsequent analyses to align with ocular development timelines and educational demand. We examined the relationship between outdoor time and reading time, and incident myopia using a Cox model, with variable selection guided by both prior literature and backward elimination based on the Bayesian information criterion, which imposes a stronger penalty on model complexity and is more conservative and consistent in large samples. We also estimated the adjusted hazard ratios (HRs) and 95% confidence intervals (CIs). We used the Schoenfeld residuals to verify the proportional hazards assumption. Furthermore, we plotted the adjusted Kaplan-Meier survival curves for incident myopia for different levels of outdoor time and reading time. Additionally, we evaluated the relationship between their combinations and the difference between them in relation to incident myopia.

We assessed the interaction effects on an additive scale of outdoor time and reading time using three indices, including excess relative risk due to interaction (RERI), attributable proportion (AP), and synergy index (SI) [11]. For two dichotomous risk factors A and B, HR_A+B+_ is the HR if both factors A and B are present, HR_A+B−_ is the HR if A is present and B is absent, HR_A−B+_ is the HR if A is absent and B is present. The RERI was calculated as: RERI = HR_A+B+_ − RR_A+B−_ − HR_A−B+_ + 1, where RERI = 0 indicates no interaction or exact additivity, RERI>0 indicates positive interaction, and RERI<0 indicates negative interaction. AP was calculated as: AP = RERI / HR_A+B+_, where AP = 0 indicates no interaction or exact additivity, AP>0 indicates positive interaction, and AP<0 indicates negative interaction. SI was calculated as: SI = (HR_A+B+_ − 1) / (HR_A+B−_ + HR_A−B+_ − 2), where SI = 1 indicates no interaction, SI>1 indicates positive interaction, and SI<1 indicates negative interaction.

For this purpose, we define risk factor A as the outdoor time of <2 hours per day and risk factor B as reading time of >2 hours per day. We determined the cut-point of 2 hours using the maximum rank statistic, a method that is outcome-oriented. We computed the generalised propensity score to balance potential confounders, including semesters at baseline, sex, parents’ myopia, time spent studying, time spent playing, and residential area. We derived the interaction metrics from both score-unweighted and weighted Cox models. We also performed subgroup analyses based on sex, baseline grade, and parental myopia to assess consistency and heterogeneity of associations across different strata.

We used *R*, version 4.2.2 (R Core Team, Vienna, Austria) for all analyses, and considered a two-tailed *P-*value of <0.05 as statistical significance.

## RESULTS

### Baseline characteristics and incidence of myopia

**Figure 2:**
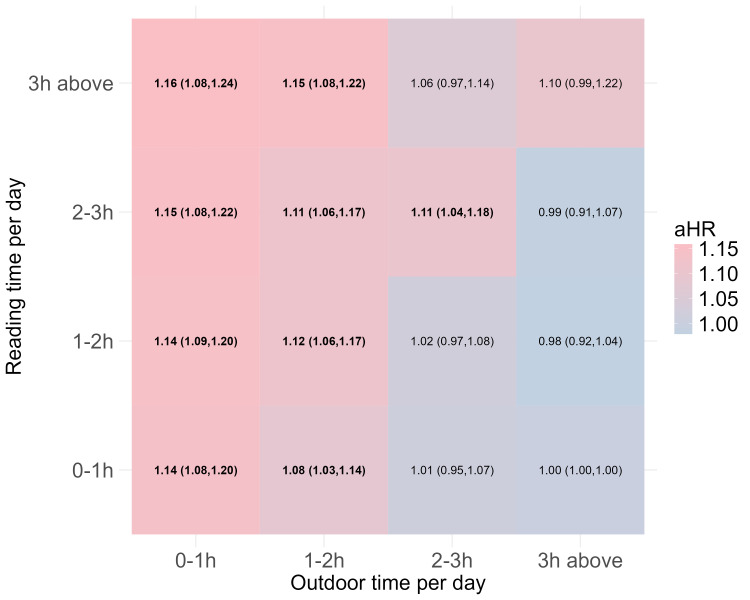
The associations between daily reading time and outdoor time, as well as their combinations with incident myopia. **Panel A.** Kaplan-Meier curve of reading time per day. **Panel B.** Kaplan-Meier curve for outdoor time per day. **Panel C.** Combinations between outdoor time and reading time on incident myopia. **Panel D.** Difference between reading time and outdoor time.

The study population comprised 219 802 children with a mean age of 8.4 (SD = 1.7) years, of whom 53.9% were boys. Between 2021–24, the cumulative incidence of myopia was 59.3% (n = 130 246). Grade four had the highest cumulative incidence of 64.0%, followed by grade five and grade six. Girls had a higher incidence (63.7%) compared to boys (55.5%). Students with a family history had a higher incidence of myopia (64.8%) compared to their counterparts (55.7%). According to the questionnaire, over 84.5% of students had lower outdoor time (<2 hours), and over 18.3% of students had the >2 hours reading time. Furthermore, the adjusted Kaplan-Meier curves suggested a median survival time of 10 semesters since entrance (**Figure 2**, Panels A and B; **Table 1**).

**Table 1:** Baseline characteristics of incident myopia*

		Incident myopia		
	**Overall**	**No**	**Yes**	***P*** *-* **value**
**All participants, n**	219 802	89 556	130 246	
**Semesters at baseline, x̄ (SD)**	5.2 (3.4)	4.9 (3.4)	5.5 (3.4)	<0.001
**Sex**				<0.001
Male	118 365 (53.9)	52 729(58.9)	65 636 (50.4)	
Female	101 437(46.1)	36 827(41.1)	64 610 (49.6)	
**SER, in D, x̄ (SD)**	0.2 (1.2)	0.4 (1.3)	0.0 (1.0)	<0.001
Missing data, n	1260	475	785	
**Family history of myopia**				<0.001
No	134 020 (61.0)	59 331 (66.3)	74 689 (57.3)	
Yes	85 782 (39.0)	30 225 (33.7)	55 557 (42.7)	
**Outdoor time per day in hours**				<0.001
0–1	56 546 (25.7)	21 784 (24.3)	34 762 (26.7)	
1–2	129 256 (58.8)	52 508 (58.6)	76 748 (58.9)	
2–3	24 392 (11.1)	10 767 (12.0)	13 625 (10.5)	
>3	9608 (4.4)	4497 (5.0)	5111 (3.9)	
**Time spent on screen play in hours**				<0.001
0–1	95 170 (43.3)	39 306 (43.9)	55 864 (42.9)	
1–2	85 204 (38.8)	34 450 (38.5)	50 754 (39.0)	
2–3	25 981 (11.8)	10 425 (11.6)	15 556 (11.9)	
>3	13 447 (6.1)	5375 (6.0)	8072 (6.2)	
**Time spent on screen study in hours**				<0.001
0–1	134 175 (61.0)	55 340 (61.8)	78 835 (60.5)	
1–2	66 285 (30.2)	26 456 (29.5)	39 829 (30.6)	
2–3	14 079 (6.4)	5629 (6.3)	8450 (6.5)	
>3	5263 (2.4)	2131 (2.4)	3132 (2.4)	
**Reading time per day in hours**				<0.001
0–1	67 183 (30.6)	28 345 (31.7)	38 838 (29.8)	
1–2	112 507 (51.2)	45 474 (50.8)	67 033 (51.5)	
2–3	29 202 (13.3)	11 544 (12.9)	17 658 (13.6)	
>3	10 910 (5.0)	4193 (4.7)	6717 (5.2)	
**Grade**				<0.001
1	62 718 (28.5)	29 485 (32.9)	33 233 (25.5)	
2	48 951 (22.3)	20 068 (22.4)	28 883 (22.2)	
3	39 623 (18.0)	14 875 (16.6)	24 748 (19.0)	
4	30 896 (14.1)	11 129 (12.4)	19 767 (15.2)	
5	22 382 (10.2)	8227 (9.2)	14 155 (10.9)	
6	15 232 (6.9)	5772 (6.4)	9460 (7.3)	
**Coarse grain**				0.005
Hardly	68 102 (31.0)	27 672 (30.9)	40 430 (31.0)	
Frequently	133 579 (60.8)	54 296 (60.6)	79 283 (60.9)	
Everyday	18 121 (8.2)	7588 (8.5)	10 533 (8.1)	
**Dessert**				0.002
Hardly	69 143 (31.5)	28 548 (31.9)	40 595 (31.2)	
Frequently	125 954 (57.3)	51 056 (57.0)	74 898 (57.5)	
Everyday	24 705 (11.2)	9952 (11.1)	14 753 (11.3)	
**Sea food**				<0.001
Hardly	85 927(39.1)	35 578 (39.7)	50 349 (38.7)	
Frequently	127 258(57.9)	51 271 (57.3)	75 987 (58.3)	
Everyday	6617(3.0)	2707 (3.0)	3910 (3.0)	
**Soda**				0.081
Hardly	189 089 (86.0)	77 169 (86.2)	111 920 (85.9)	
Frequently	29 151 (13.3)	11 727 (13.1)	17 424 (13.4)	
Everyday	1562 (0.7)	660 (0.7)	902 (0.7)	
**Salt, in g/per day**				0.100
<4	44 503 (20.2)	18 295 (20.4)	26 208 (20.1)	
4–6	171 648 (78.1)	69 813 (78.0)	101 835 (78.2)	
>6	3651 (1.7)	1448 (1.6)	2203 (1.7)	
**Fruits**				<0.001
Hardly	4980 (2.3)	2134 (2.4)	2846 (2.2)	
Frequently	76 211 (34.7)	31 709 (35.4)	44 502 (34.2)	
Everyday	138 611 (63.1)	55 713 (62.2)	82 898 (63.6)	
**Vegetables**				<0.001
Hardly	8860 (4.0)	3739 (4.2)	5121 (3.9)	
Frequently	76 177 (34.7)	31 419 (35.1)	44 758 (34.4)	
Everyday	134 765 (61.3)	54 398 (60.7)	80 367 (61.7)	

SD – standard deviation, SER – spherical equivalent refraction, x̄ – mean

*All values are presented as numbers (percentages) unless specified otherwise.

†*P*-values for categorical variables are from the Pearson χ^2^ test, and from the Wilcoxon rank sum test for continuous variables.

### The associations between environmental factors and incident myopia

After adjusting for other covariates, increased outdoor time of 1–2 hours (HR = 0.97; 95% CI = 0.95, 0.98), 2–3 hours (HR = 0.90; 95% CI = 0.88, 0.92), and >3 hours (HR = 0.86; 95% CI = 0.84, 0.89) were associated with a lower risk of myopia (**Table 2**). Conversely, prolonged reading time of 1–2 hours (HR = 1.02; 95% CI = 1.01, 1.03), 2–3 hours (HR = 1.03; 95% CI = 1.01, 1.05), and >3 hours (HR = 1.05; 95% CI = 1.03, 1.08) were associated with a higher risk of myopia. Furthermore, the combinations of outdoor time and reading time suggested that the onset of myopia would significantly increase once reading time was higher than or equal to the outdoor time and the differences were also linked to the increased HRs (**Figure 2**, Panels C and D).

**Table 2:** Association between outdoor time and reading time with incident myopia

	Participants	Myopia events	HR (95% CI)	*P*-value
Outdoor time per day in hours*				
0–1	56 546	34 762	ref	
1–2	129 256	76 748	0.965 (0.952, 0.977)	<0.001
2–3	24 392	13 625	0.901 (0.883, 0.920)	<0.001
>3	9608	5111	0.864 (0.839, 0.890)	<0.001
Reading time per day*				
0–1	67 183	38 838	ref	
1–2	112 507	67 033	1.020 (1.007, 1.033)	0.002
2–3	29 202	17 658	1.030 (1.011, 1.049)	0.002
>3	10 910	6717	1.052 (1.025, 1.080)	<0.001

CI – confidence interval, HR – hazard ratio, ref – reference

*Adjusted by semester at baseline, sex, parental myopia, time spent on screen for study, time spent on screen for playing, and residential areas.

### The additive interaction between outdoor time and reading time

Based on the Cox model, we calculated the maximally selected rank statistics for outdoor time and reading time, which suggested 2 hours were the best cut-offs. Thus, we categorised the outdoor time and reading time into two levels. The results indicated that the outdoor time and reading time had significant interactive effects on incident myopia with the RERI of −0.04 (95% CI = −0.08, 0.00), AP of −0.04 (95% CI = −0.07, 0.00), and SI of 0.71 (95% CI = 0.50, 0.93) (**Table 3**).

**Table 3:** Interaction effects between outdoor and reading time

	Unweighted				Weighted			
	**HR (95% CI)**	**RERI (95% CI)**	**AP (95% CI)**	**SI (95% CI)**	**HR (95% CI)**	**RERI (95% CI)**	**AP (95% CI)**	**SI (95% CI)**
**OT>2 h × RT 0–2h**	ref	−0.04 (−0.08, −0.01)	−0.04 (−0.07, −0.01)	0.73 (0.58, 0.93)	ref	−0.04 (−0.08, 0.00)	−0.04 (−0.07, 0.00)	0.71 (0.50, 0.93)
**OT>2 h × RT>2 h**	1.06 (1.02, 1.09)				1.05 (1.01, 1.09)			
**OT 0–2 h × RT 0–2 h**	1.11 (1.09, 1.13)				1.09 (1.07, 1.11)			
**OT 0–2 h × RT>2 h**	1.12 (1.10, 1.15)				1.10 (1.07, 1.12)			

AP – attributable proportion, CI – confidence interval, HR – hazard ratio, OT – outdoor time, ref – reference, RERI – relative risk due to interaction, RT – reading time, SI – synergy index

**Figure 3:**
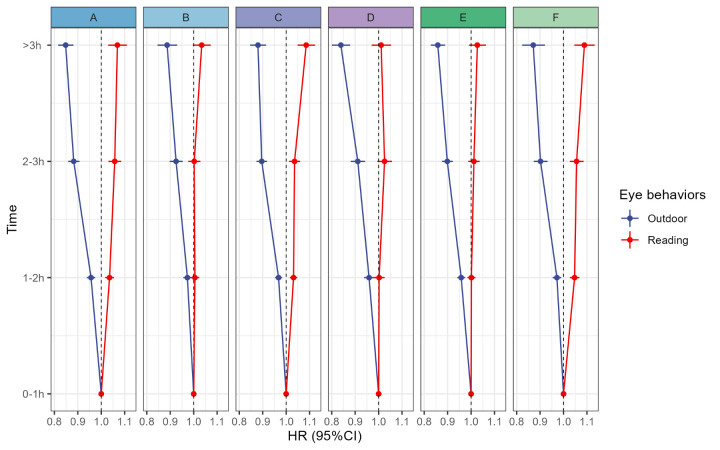
The differences in associations between outdoor time and reading, along with their interaction in different groups. **Panel A.** Outdoor and reading time among boys, girls, grade ≤3, grade ≥3, those without a myopic parent, and those with a myopic parent. **Panel B.** Difference in reading and outdoor time among boys, girls, grade ≤3, grade ≥3, those without a myopic parent, and those with a myopic parent. **Panel C.** Interaction indices of outdoor and reading time among boys, girls, grade ≤3, grade ≥3, those without a myopic parent, and those with a myopic parent.

The subpopulation analyses suggested that outdoor time was associated with an increased risk of myopia onset in all groups. However, reading time was significantly associated with myopia in boys, those in grades from 1 to 3, and those with myopic parents (*P* < 0.05) (**Figure 3**, Panel A). The differences between them were positively related to the risk of myopia onset (**Figure 3**, Panel B). The interactive effects on the additive scale were significant in boys, those in grades 1 to 3, and students with myopic parents (**Figure 3**, Panel C).

## DISCUSSION

To our knowledge, we are the first to comprehensively evaluate the interaction effects of outdoor time and reading time among almost 200 000 primary school students. Our findings demonstrate that these factors are not only independently related to the onset of myopia but also interact with each other negatively, meaning sufficient outdoor time may buffer the risks of prolonged reading, especially in boys, those in grades 1–3, and those with myopic parents.

Using non-cycloplegic refraction, we found that the three-year cumulative incidence of myopia among primary school students in Tianjin was 59.3%, aligning with previous school-based cohort studies in China [12]. While non-cycloplegic measurement may overestimate the true incident myopia in primary school students due to accommodation interference, age-related variability, and subjective response bias [13]. It was still pragmatic for large screening settings, as its time efficiency and its sensitivity combined with uncorrected visual acuity [14]. Moreover, pseudo-myopia was also considered an independent risk for myopia onset [15], which could serve as an agent for myopia. In addition, the higher incidence among older grades likely reflects education onward and increased academic burden for students, instead of age only [16]. The contrast between 18.3% of students exceeding two hours of daily reading and over 80% spending less than two hours outdoors emphasises insufficient outdoor exposure as a primary concern.

In this study, both outdoor time and reading time were self-reported by parents, which may introduce recall and social desirability bias. To reduce this bias, we developed the questionnaire based on validated instruments used in previous studies and instructed the participants to report behaviours over the recent seven days. However, self-reported measures of outdoor time and reading time still inevitably led to misclassification, potentially inflating or attenuating the observed associations. Previous studies suggested that overestimation of outdoor is common among parents [17,18], which could bias the results towards the null hypothesis. Thus, the observed protective effects may be underestimated.

Consistent with previous literature, our findings suggest that outdoor time was inversely associated with myopia onset, likely mediated by light-induced dopamine release that inhibits axial elongation [18–23]. The observed buffering effect of outdoor time on prolonged reading supports this mechanistic link. Although previous studies have questioned the association between near work and myopia [24–26] – often because near work was not consistently defined and measures of near viewing behaviour varied – our results suggest that reading time alone may have a modest effect, which becomes more pronounced when combined with limited outdoor exposure.

We identified a negative additive interaction between reading time and outdoor time on the risk of incident myopia. This indicates that the combined influence of high reading time and low outdoor time on myopia development is smaller than the sum of their individual effects. The possible reasons were exposure to outdoor light, which is hypothesised to stimulate retinal dopamine release, which inhibits axial elongation – a known anatomical change associated with myopia [27,28]. Thus, among children with prolonged near-work exposure, sufficient outdoor time might attenuate the progression toward myopia. Similar patterns have been observed in large population studies where increased outdoor exposure was shown to reduce myopia risk, despite high academic demands [29,30]. On the other hand, both AP and RERI suggested a relatively modest effect size. From a clinical perspective, this interaction may be considered negligible at the individual level. However, given the high baseline prevalence of myopia and the large number of children exposed to both risk factors, even small interaction effects can translate into meaningful risk reductions at the population level.

Subgroup analyses revealed that the interaction between reading time and outdoor time was significantly associated with an increased risk of incident myopia in specific populations – boys, children in lower grades, and those with myopic parents. These findings suggest that the combined influence of high reading exposure and limited outdoor activity may have a disproportionate impact on these vulnerable subgroups. One plausible explanation is that younger children (*i.e.* those in lower grades) are in a more plastic phase of ocular development, during which environmental exposures have a stronger influence on eye growth [28]. In boys, the heightened risk may reflect behavioural or physiological differences, such as lower compliance with visual hygiene practices or greater sensitivity to environmental stressors [31]. Additionally, children with a genetic predisposition may have a lower biological threshold for environmental triggers, and thus, near work behaviours like excessive reading may more readily translate into pathological axial elongation [32]. These findings underscore the need for targeted interventions to prevent myopia. Interventions should be age- and sex-sensitive and should prioritise children with a family history of myopia by ensuring adequate daily outdoor exposure and managing reading workloads from an early age.

Interestingly, we found that the second semester of grades 4–7 represents a critical window for myopia onset, suggesting a temporal clustering of incident cases during this developmental phase. This pattern likely reflects the increased academic demands and intensified near-work characteristic of these school years, particularly in preparation for entrance exams and curriculum transitions. Additionally, these grades often coincide with reduced outdoor activity due to heavier workloads, extracurricular tutoring, and parental emphasis on academic achievement.

The second-semester effect may further be influenced by seasonal factors, such as shorter daylight hours and colder weather, which limit outdoor exposure and exacerbate near-work burden. These findings underscore the importance of targeted interventions during these high-risk periods, including implementing structured outdoor activity programmes and regulating reading and screen time, especially in the months when students are most vulnerable to behavioural shifts that predispose them to myopia.

Our findings underscore the pressing need to increase outdoor activity and moderate reading time, particularly among children at heightened risk of myopia and in the critical windows for myopia onset. Given the well-established protective effect of outdoor exposure, integrating structured outdoor play into daily routines should be prioritised in both schools and communities. Concurrently, reducing prolonged near work may further diminish the likelihood of myopia onset.

Public health initiatives should promote a balanced lifestyle by encouraging children – particularly younger children, boys, and those with a family history of myopia – to spend at least two hours per day outdoors while limiting visually demanding activities during the remaining time. Future strategies for myopia prevention and health promotion must also address challenges in resource-limited settings, where safety concerns, urban infrastructure deficits, and funding shortages may hinder implementation. To overcome these barriers, policies should aim to strengthen local infrastructure through the development of health facilities and community recreational spaces and empower communities via health education and capacity-building initiatives. Financial assistance, such as targeted subsidies and insurance support, can alleviate economic constraints. Public awareness campaigns are essential to promote healthy visual behaviours from an early age. Moreover, intersectoral collaboration among the health, education, and urban planning sectors is crucial for creating environments that promote eye health. The deployment of mobile health services can further expand reach in underserved regions. Ultimately, sustained policy commitment and strategic investment are crucial to reducing health inequities and ensuring equitable access to effective myopia prevention and eye care services across all population groups.

While we comprehensively explored the combined and interaction effects of time spent outdoors and reading based on a large real-world data set, some limitations still exist. First, we defined myopia by uncorrected visual acuity and non-cycloplegic refraction, considering the large number of participants and the feasibility of the survey, which may have overestimated the prevalence of myopia. Second, the outdoor time and reading time were self-reported via an online questionnaire; the recall bias was inevitable, potentially underestimating the protective effects. Future studies should consider integrating objective measurements (*e.g.* wearable light sensors or digital time-tracking) to validate exposure estimates and reduce bias from self-reports. Third, residual confounding from unmeasured factors (*e.g.* socioeconomic status, urban green space access, parental education) may exist, and we did not account for the dynamic behaviour trajectory in the analysis. Future studies should incorporate this information to refine causal inference.

## CONCLUSIONS

This large-scale cohort study highlights both the independent and joint effects of environmental behaviours on childhood myopia. Promoting sufficient outdoor exposure may significantly mitigate the adverse effects of prolonged reading – especially in vulnerable populations. Our findings provide evidence to inform public health strategies for myopia prevention, particularly during the early school years.

## References

[1] Holden BA Fricke TR Wilson DA Jong M Naidoo KS Sankaridurg P Global Prevalence of Myopia and High Myopia and Temporal Trends from 2000 through 2050. Ophthalmology. 2016;123:1036–42. 10.1016/j.ophtha.2016.01.006 26875007

[2] Tuo Y Zhang G Yi H Vision for the future: pioneering strategies in China’s battle against myopia. Eye (Lond). 2024;38:3042–4. 10.1038/s41433-024-03250-7 39026097 PMC11544143

[3] Ma Y Wen Y Zhong H Lin S Liang L Yang Y Healthcare utilization and economic burden of myopia in urban China: A nationwide cost-of-illness study. J Glob Health. 2022;12:11003. 10.7189/jogh.12.11003 35356656 PMC8934110

[4] Lipson MJ Boland B McAlinden C Vision-related quality of life with myopia management: A review. Cont Lens Anterior Eye. 2022;45:101538. 10.1016/j.clae.2021.101538 34802915

[5] Lu C Miao Y Yao X Wang Z Wei R Du B Socioeconomic disparities and green space associated with myopia among Chinese school-aged students: A population-based cohort study. J Glob Health. 2024;14:04140. 10.7189/jogh.14.04140 38898796 PMC11187523

[6] Mountjoy E Davies NM Plotnikov D Smith GD Rodriguez S Williams CE Education and myopia: assessing the direction of causality by mendelian randomisation. BMJ. 2018;361:k2022. 10.1136/bmj.k2022 29875094 PMC5987847

[7] Lingham G Mackey DA Lucas R Yazar S How does spending time outdoors protect against myopia? A review. Br J Ophthalmol. 2020;104:593–9. 10.1136/bjophthalmol-2019-314675 31722876

[8] He M Xiang F Zeng Y Mai J Chen Q Zhang J Effect of Time Spent Outdoors at School on the Development of Myopia Among Children in China: A Randomized Clinical Trial. JAMA. 2015;314:1142–8. 10.1001/jama.2015.10803 26372583

[9] He X Sankaridurg P Wang J Chen J Naduvilath T He M Time Outdoors in Reducing Myopia: A School-Based Cluster Randomized Trial with Objective Monitoring of Outdoor Time and Light Intensity. Ophthalmology. 2022;129:1245–54. 10.1016/j.ophtha.2022.06.024 35779695

[10] Morgan IG Wu PC Ostrin LA Tideman JWL Yam JC Lan W IMI Risk Factors for Myopia. Invest Ophthalmol Vis Sci. 2021;62:3. 10.1167/iovs.62.5.3 33909035 PMC8083079

[11] Knol MJ VanderWeele TJ Groenwold RHH Klungel OH Rovers MM Grobbee DE Estimating measures of interaction on an additive scale for preventive exposures. Eur J Epidemiol. 2011;26:433–8. 10.1007/s10654-011-9554-9 21344323 PMC3115067

[12] Wang SK Guo Y Liao C Chen Y Su G Zhang G Incidence of and Factors Associated With Myopia and High Myopia in Chinese Children, Based on Refraction Without Cycloplegia. JAMA Ophthalmol. 2018;136:1017–24. 10.1001/jamaophthalmol.2018.2658 29978185 PMC6142978

[13] Wilson S Ctori I Shah R Suttle C Conway ML Systematic review and meta-analysis on the agreement of non-cycloplegic and cycloplegic refraction in children. Ophthalmic Physiol Opt. 2022;42:1276–88. 10.1111/opo.13022 35913773 PMC9804580

[14] Sankaridurg P He X Naduvilath T Lv M Ho A Smith E Comparison of noncycloplegic and cycloplegic autorefraction in categorizing refractive error data in children. Acta Ophthalmol. 2017;95:e633–40. 10.1111/aos.13569 29110438 PMC5698763

[15] Sun W Yu M Wu J Han X Jan C Song J Pseudomyopia as an independent risk factor for myopia onset: a prospective cohort study among school-aged children. Br J Ophthalmol. 2024;108:873–8. 10.1136/bjo-2022-322330 37541767 PMC11137461

[16] Ding X Morgan IG Hu Y Tang X Zhang J Guo L The Causal Effect of Education on Myopia: Evidence That More Exposure to Schooling, Rather Than Increased Age, Causes the Onset of Myopia. Invest Ophthalmol Vis Sci. 2023;64:25. 10.1167/iovs.64.4.25 37083951 PMC10132316

[17] Wen L Cao Y Cheng Q Li X Pan L Li L Objectively measured near work, outdoor exposure and myopia in children. Br J Ophthalmol. 2020;104:1542–7. 10.1136/bjophthalmol-2019-315258 32075819 PMC7587221

[18] Chen J Wang J Qi Z Liu S Zhao L Zhang B Smartwatch Measures of Outdoor Exposure and Myopia in Children. JAMA Netw Open. 2024;7:e2424595. 10.1001/jamanetworkopen.2024.24595 39136948 PMC11322842

[19] Dhakal R Shah R Huntjens B Verkicharla PK Lawrenson JG Time spent outdoors as an intervention for myopia prevention and control in children: an overview of systematic reviews. Ophthalmic Physiol Opt. 2022;42:545–58. 10.1111/opo.12945 35072278 PMC9305934

[20] Stone RA Lin T Laties AM Iuvone PM Retinal dopamine and form-deprivation myopia. Proc Natl Acad Sci U S A. 1989;86:704–6. 10.1073/pnas.86.2.704 2911600 PMC286542

[21] Munteanu T Noronha KJ Leung AC Pan S Lucas JA Schmidt TM Light-dependent pathways for dopaminergic amacrine cell development and function. eLife. 2018;7:e39866. 10.7554/eLife.39866 30403373 PMC6221543

[22] Feldkaemper M Schaeffel F An updated view on the role of dopamine in myopia. Exp Eye Res. 2013;114:106–19. 10.1016/j.exer.2013.02.007 23434455

[23] Deng L Gwiazda J Thorn F Children’s refractions and visual activities in the school year and summer. Optom Vis Sci. 2010;87:406–13. 10.1097/OPX.0b013e3181da8a85 20375747 PMC3329178

[24] Lanca C Yam JC Jiang WJ Tham YC Hassan Emamian M Tan CS Near work, screen time, outdoor time and myopia in schoolchildren in the Sunflower Myopia AEEC Consortium. Acta Ophthalmol. 2022;100:302–11. 10.1111/aos.14942 34142457

[25] Huang HM Chang DS Wu PC The Association between Near Work Activities and Myopia in Children-A Systematic Review and Meta-Analysis. PLoS One. 2015;10:e0140419. 10.1371/journal.pone.0140419 26485393 PMC4618477

[26] Guan H Yu NN Wang H Boswell M Shi Y Rozelle S Impact of various types of near work and time spent outdoors at different times of day on visual acuity and refractive error among Chinese school-going children. PLoS One. 2019;14:e0215827. 10.1371/journal.pone.0215827 31026279 PMC6485919

[27] Rose KA Morgan IG Ip J Kifley A Huynh S Smith W Outdoor Activity Reduces the Prevalence of Myopia in Children. Ophthalmology. 2008;115:1279–85. 10.1016/j.ophtha.2007.12.019 18294691

[28] French AN Ashby RS Morgan IG Rose KA Time outdoors and the prevention of myopia. Exp Eye Res. 2013;114:58–68. 10.1016/j.exer.2013.04.018 23644222

[29] Guggenheim JA Northstone K McMahon G Ness AR Deere K Mattocks C Time outdoors and physical activity as predictors of incident myopia in childhood: a prospective cohort study. Invest Ophthalmol Vis Sci. 2012;53:2856–65. 10.1167/iovs.11-9091 22491403 PMC3367471

[30] Li SM Li SY Kang MT Zhou Y Liu LR Li H Near Work Related Parameters and Myopia in Chinese Children: the Anyang Childhood Eye Study. PLoS One. 2015;10:e0134514. 10.1371/journal.pone.0134514 26244865 PMC4526691

[31] Sherwin JC Reacher MH Keogh RH Khawaja AP Mackey DA Foster PJ The Association between Time Spent Outdoors and Myopia in Children and Adolescents. Ophthalmology. 2012;119:2141–51. 10.1016/j.ophtha.2012.04.020 22809757

[32] Lin Y Jiang D Li C Huang X Xiao H Liu L Interactions between genetic variants and near-work activities in incident myopia in schoolchildren: a 4-year prospective longitudinal study. Clin Exp Optom. 2023;106:303–10. 10.1080/08164622.2021.2024070 35021948

